# The Orexin receptors: Structural and anti-tumoral properties

**DOI:** 10.3389/fendo.2022.931970

**Published:** 2022-07-28

**Authors:** Alain Couvineau, Pascal Nicole, Valérie Gratio, Thierry Voisin

**Affiliations:** INSERM UMR-S1149/Center of Research on Inflammation (CRI), Université Paris Cité, Team “From Inflammation to Cancer in Digestive Diseases”, DHU UNITY, Paris, France

**Keywords:** G protein-coupled receptor(GPCR), protein structure, pharmacology, structure-function relationship, cancer, Orexins, Orexin receptor

## Abstract

At the end of the 20th century, two new neuropeptides (Orexin-A/hypocretin-1 and Orexin-B/hypocretins-2) expressed in hypothalamus as a prepro-orexins precursor, were discovered. These two neuropeptides interacted with two G protein-coupled receptor isoforms named OX1R and OX2R. The orexins/OX receptors system play an important role in the central and peripheral nervous system where it controls wakefulness, addiction, reward seeking, stress, motivation, memory, energy homeostasis, food intake, blood pressure, hormone secretions, reproduction, gut motility and lipolysis. Orexins and their receptors are involved in pathologies including narcolepsy type I, neuro- and chronic inflammation, neurodegenerative diseases, metabolic syndrome, and cancers. Associated with these physiopathological roles, the extensive development of pharmacological molecules including OXR antagonists, has emerged in association with the determination of the structural properties of orexins and their receptors. Moreover, the identification of OX1R expression in digestive cancers encompassing colon, pancreas and liver cancers and its ability to trigger mitochondrial apoptosis in tumoral cells, indicate a new putative therapeutical action of orexins and paradoxically OXR antagonists. The present review focuses on structural and anti-tumoral aspects of orexins and their receptors.

## Introduction

The discovery of the orexins system is relatively recent since its identification dates back to the late 90s (1, 2). Orexins also termed hypocretins were simultaneously discovered by two independent groups in 1998 (1, 2). The term hypocretin was derived from in « hypo » from hypothalamus which corresponded to the production location of these peptides and « cretin » because these peptides have a light sequence homology with the secretin hormone (1), whereas the term « orexin » comes from the Greek word « orexis » meaning « appetite » based on the first observations indicating that orexins regulated food intake (2). For further clarity, the term « orexins » will be preferably chosen in the present review, assuming that nomenclature recommendations reserved the term « orexins » to peptides and proteins. Orexin peptides are divided in two isoforms termed orexin-A (OxA or hypocretin-1) and orexin-B (OxB or hypocretin-2) produced by the same precursor in the hypothalamus, the prepro-orexin which is encoded by HCRT (hypocretin neuropeptide precursor) gene located on chromosome 17 (2). OxA and OxB sequences are highly conserved among mammalian species in which orexins have been identified encompassing human, mouse, rat, pig (3). However, OxB presented weak variation in mammals (3). Orexins are also expressed in birds, reptiles, amphibians and fishes where the sequences are similar whereas more variability was observed in particular in the N-terminal domain (3–5). Conversely, orexins-like genes have not been identified in invertebrates (3).

**Figure 1:**
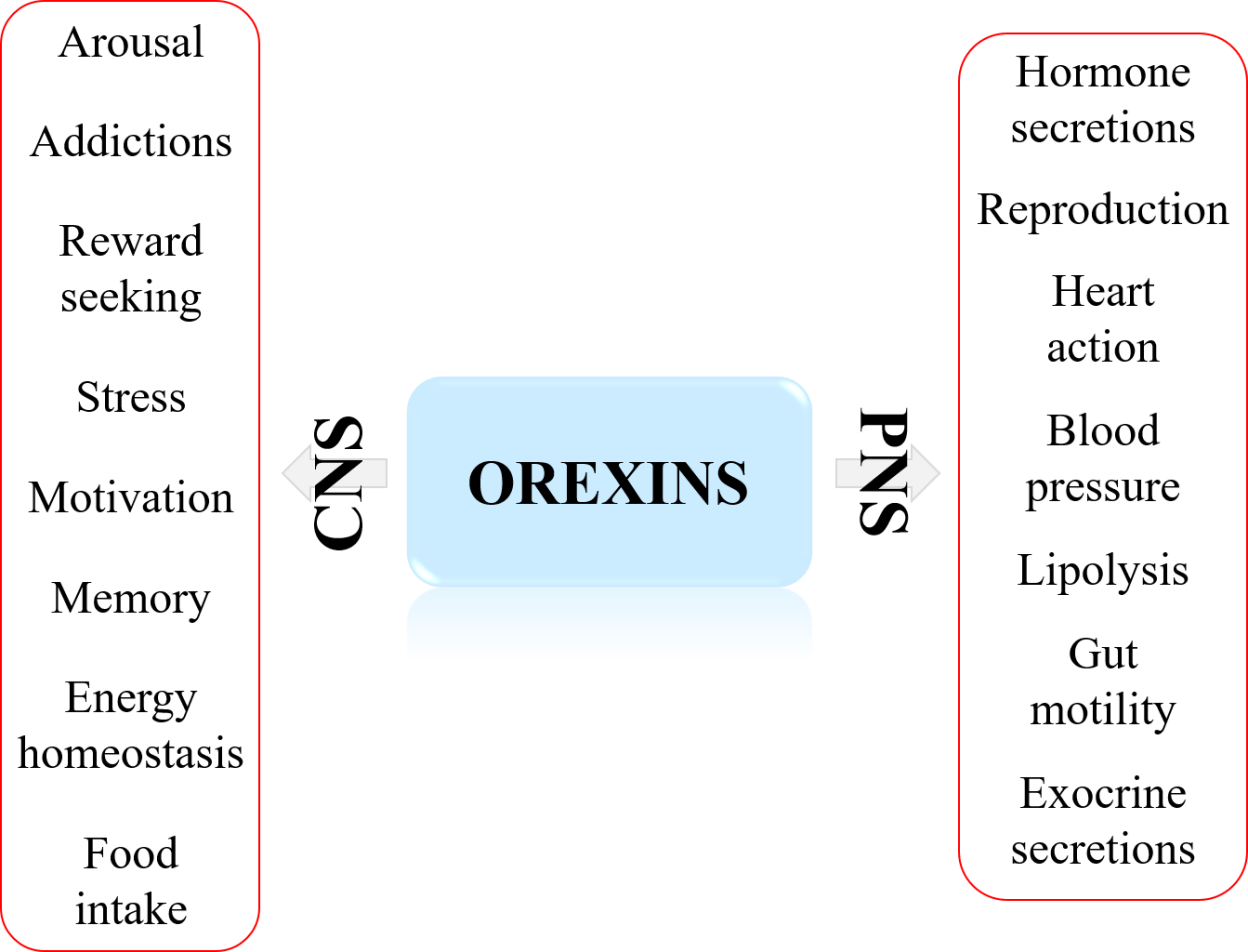
Major biological actions of orexins in central nervous system (CNS) and peripheral nervous system (PNS).

The major physiological action (**Figure 1**) in central nervous system (CNS) of the orexins/OXR system, widely studied for over 20 years, was to maintain wakefulness (6, 7). The main pathology associated to this action was the narcolepsy type I characterized by excessive daytime sleepiness (EDS) associated to Rapid Eye Movement (REM) symptoms including cataplexy, hallucinations, sleep paralysis and fragmentation of night sleep (7, 8). This rare neurological disorder was caused by the loss of orexin neurons leading to the absence of orexins secretion (7) but in dog, this disorder was related to orexin receptor mutation (9). It should be noted that obesity was more prevalent in patient with narcoleptic type I disorder than in the general population (10). These observations suggested the existence of a putative pathogenic link between higher BMI (Body Mass Index) and alteration of orexin signaling in narcoleptic patients (10). In addition, orexins were also involved in other multiple physiological actions in CNS (**Figure 1**) encompassing energy homeostasis, drug addiction, food consumption, motivation, and reward seeking (11, 12). Beyond these CNS actions, orexins and their receptors had biological impacts in peripheral nervous system (PNS). Although less investigated, the role of orexin in PNS (**Figure 1**) revealed actions on neuroendocrine functions, blood pressure, metabolism, reproductive functions, energy balance and gastrointestinal motility (13).

The present review will summarize a large overview ranging from the orexins/OX receptor identification, their structure and pharmacology to orexin peripheral physiopathological actions mainly illustrating by anti-tumoral properties of orexins and, curiously, of receptor antagonists where their impact could be deciphered by the OX1R structure-function relationship analysis.

## Orexins structures

**Figure 2:**
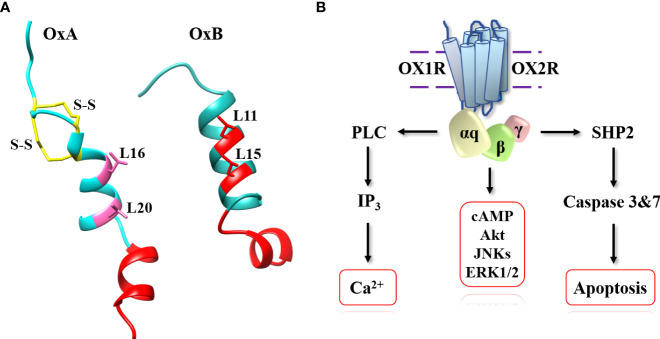
OxA and OxB structures **(A)** and schematic representation of signaling pathways activated by orexins. **(A)** (left): OxA structure (light blue), disulfide bridges (yellow), crucial domain involved in OxA activity (red) and putative leucine residues involved in OxA activity (pink). Panel A (right): OxB structure (light sea green) and crucial domain and important leucine residues involved in OxB activity (red). **(B)**: phospholipase C (PLC), inositol-1,4,5-trisphosphate (IP_3_), protein kinase B (Akt), c-JUN N-terminal kinases (JNK), extracellular signal-regulated kinases (Erk1/2) and tyrosine-protein phosphatase non-receptor type 11 (SHP2).

The two neuropeptides, OxA and OxB are produced from prepro-orexin, a precursor of 131 residues, by proteolytic cleavage at consensus sites (G-R/K-R/K) (2, 3). In mammals, OxA has a length of 33 residues and OxB, a length of 28 residues (14). The two neuropeptides are C-terminal amidated (14). OxA contains a pyroglutamyl modified residue at N-terminal position and two intrachain disulfide bridges (**Figure 2**) formed by 4 cysteine residues (C6-C12 and C7-C14, respectively) (15). These two disulfide bridges were highly conserved in all species (3). In contrast, OxB does not contain disulfide bridges or modified residues. As mentioned in the introduction, both orexins have some similarities in C-terminal regions with secretin peptide hormone. However, no cross-reaction between secretin and orexins receptors in mammals has been identified and no evidence for a common ancestral origin between these peptides has been demonstrated in vertebrates (16, 17). Structure-activity relationship (SAR) studies of OxA revealed that disulfide bridges presence in the peptide was not essential for its full activity (18). The deletion of the N-terminal domain residues of OxA between positions 2 and 15 had no real impact on the peptide activity (18, 19). In contrast, the deletion of the central domain between residues 15 and 19 strongly reduced the peptide activity (18–20). The N-terminal fragment (1–13) did not have any role in peptide activity while the OxA C-terminal domain (sequence 20 to 33) was very important for its activity (**Figure 2**) (18). In parallel, the analysis of the OxB SAR shown that the deletion of the first 6 residues of the peptide had no impact on its activity whereas alanine scanning of the peptide revealed that L^11^, L^15^, A^22^, G^24^, I^25^, L^26^ and M^28^ residues were essential for the peptide activity (18, 21). It should be noted that these residues are mainly located in the OxB C-terminal moiety indicating that the C-terminal domain of OxB and also OxA (**Figure 2**) which was crucial their activities could play a major role in receptor binding sites presumably by interaction into orthosteric sites of OX receptors (21–23). Moreover, the importance of L11 and L15 (**Figure 2**) present in N-terminal/central part of OxB sequence could interact with OX1R extracellular domains (21). Although not clearly defined, L16 and L20 residues could play the same role in OxA (**Figure 2**). A review of various single-nucleotide polymorphisms (SNPs) present in orexigenic neuropeptide sequences including OxA and OxB in BMI context revealed that SNPs were present along OxA and OxB sequences (24). Some of these SNPs corresponding to missense mutation, particularly located in the C-terminal domain of OxA and OxB could have a negative impact on the peptide activity (24). In 1999, the first identification of OxB structure in solution based on 2-dimensional nuclear magnetic resonance (NMR) was established (25). The preliminary analysis by circular dichroism spectrum had demonstrated the presence of a significant population of α-helix (25). NMR assignments associated to dynamical simulated annealing calculations revealed the existence of two α-helices (**Figure 2**) with orientation of about 60-80° connected by a kink region (25). This structure was confirmed by NMR analysis of OxB in sodium dodecyl sulfate micellar solution revealing two α-helices between residues 7 to 18 and residues 22 to 26 connected by a flexible loop (**Figure 2**) (26). Up to now, the structure of OxA has not been characterized but a 3D model obtained by homology modeling based on the OxB structure has been produced (27). This model was similar to the OxB structure characterized by two α-helices connected by a short linker (**Figure 2**). However, the presence of two disulfide bridges in OxA reduced the flexibility of N-terminal domain of the peptide (27).

## OX receptors structures and pharmacology

OxA and OxB interact with two receptors named orexin-receptor type 1 (OX1R) and orexin-receptor type 2 (OX1R) which belong to the G protein-coupled receptor (GPCR) family of class A also termed rhodopsin-like receptors (3, 28). OX1R was encoded by the HCRTR1 (hypocretin receptor type 1) gene located on chromosome 1 and OX2R was encoded by the HCRTR2 (hypocretin receptor type 1) gene located on chromosome 6 (1, 2). These two receptors shared 64% of identity sequence (27) and they also have a sequence identity (about 30%) with other class A GPCRs such as neuropeptide FF receptor (29). OX1R and OX2R bind OxA with the same affinity whereas OxB has a best affinity for OX2R than for OX1R (30). Both OX1R and OX2R were expressed in mammals while only OX2R was identified in non-mammals (3). These observations suggested that OX2R represents the ancestral receptor form whereas OX1R would have evolved during the mammals’ evolution, from OX2R by gene duplication (3, 6). Moreover, emergence of OX1R could be associated to its more complex role in mammal pathophysiology (3, 6).

It was not until the 2000s that the first pharmacologic compounds named SB-334867-A/(1-(2-Methylbenzoxazol-6-yl)-3-[1,5]napthyridin-4-yl-urea hydrochloride), an OX1R antagonist, was developed by GlaxoSmithKline Pharmaceuticals (31). One of the major roles of orexins being to regulate sleep, the development by academic and pharmaceutical laboratories of antagonists represented/represents a strong challenge to treat insomnia (32). In this context a lot of antagonists have been designed to control wake-sleep cycles (33, 34). These antagonists were classified into two groups defined as SORAs (single orexin-receptor antagonists) including SORA1s (selective OX1R antagonist) as SB-334867 (31) and SORA2s (selective OX2R antagonist) as seltorexant (JNJ-42847922) (35), and DORAs (dual orexin-receptor antagonists) as almorexant which interacted similarly with OX1R and OX2R (36). To date, three antagonists named suvorexant (37), lemborexant (38) and daridorexant (39) which are DORAs, were approved by the U.S. Food & Drug Administration (FDA) and prescribed in insomnia treatment. While the development of orexin receptors antagonists has been successful, few orexin receptor agonists have been designed related to the weak interest of the pharmaceutical industries for such compounds (40). Two types of molecules have been investigated encompassing modified peptides and non-peptide molecules. The modification of OxA sequence by aminoacid substitution such as [Ala11, D-Leu15] orexin-B (41) which displayed selectivity for OX2R, has been firstly investigated. The first OX2R selective non-peptide molecule, YNT185 developed in 2015, reduced cataplectic attacks in orexin peptide-deficient mice (42). More recently, the high OX2R selective compound, TAK925, has been designed by Yukitake *et al.* (43). TAK925 increased the wake and reduced the sleep-wake cycles (44). The same group has developed an orally available OX2R agonist, TAK994 (45). At this time, no OX1R selective agonists have been designed.

**Figure 3:**
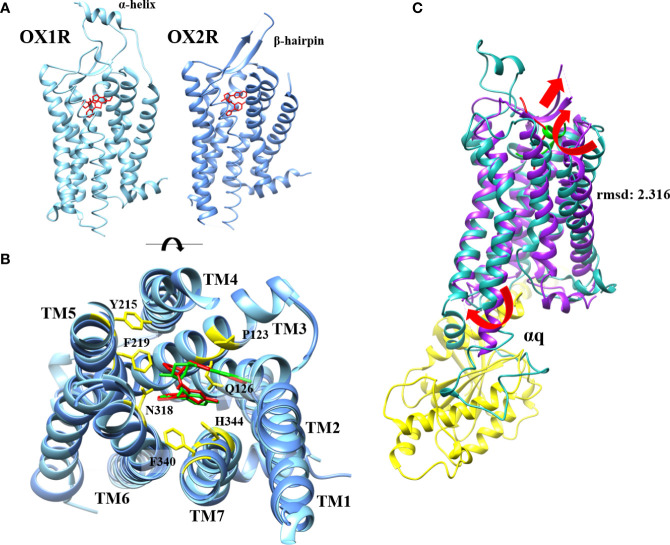
OX1R-suvorexant and OX2R-suvorexant structures **(A)**, orthosteric site of OX1R-suvorexant and OX2R-suvorexant **(B)** and comparison between OX2R-OxB structure obtained by cryo-EM and OX1R-OxB 3D model obtained by homology modeling associated to molecular dynamic simulation **(C)**. **(A)** (left): OX1R X-ray structure (light blue) bounded to suvorexant (red). **(A)** (right): OX2R X-ray structure (cornflower blue) bounded to suvorexant (red). Panel B: Orthosteric site of OX1R (light blue) bounded to suvorexant (red) and OX2R (cornflower blue) bounded to suvorexant (green). OX1R and OX2R residues involved in suvorexant interactions (yellow). Panel C: OX2R cryo-EM structure (purple) bounded to OxB (red) and OX1R 3D model (light sea green) bounded to C-terminal domain of OxB (green). αq subunit of mini-Gq protein (yellow). Red arrows indicate TM movements in active form.

Less than twenty years after the discovery of OX1R and OX2R, the first X-ray structure of OX2R was obtained in 2015 (46). The determination of GPCRs X-ray structure was initiated by Kobilka and Lefkowitz’s work (Nobel Prizes, 2012) allowing relatively quickly, the acquisition of a large number of GPCR structures (47). The first structure determination was performed on OX2R complexed to the suvorexant antagonist molecule (45). This structure (**Figure 3**) composed by a seven transmembrane (TM) fold resembles to other GPCRs (45). For instance, the root-mean-square distance (rmsd) between OX2R and β_2_-AR was very close (2.2 Å) although a very low homology sequence between these two receptors was observed (45). It should be noted that the highly conserved E/DRY, sequence motif which played as an inhibitory interaction network in class A GPCRs, was replaced by DRWY sequence motif in OX2R (45). The orthosteric site occupied by the suvorexant antagonist was open to extracellular space *via* a solvent-accessible channel (45). The presence of a β-hairpin structure (**Figure 3**) was identified in extracellular loop (ECL) 2 (48). An electrostatic network stabilized the extracellular sides of TMs. Suvorexant interacted with all TMs excepted TM1 (48). Hydrogen bonds mediated by water molecules forming electrostatic bridges with N224^6.55^ and H350^7.39^ stabilized interactions between suvorexant and binding pocket (**Figure 3**) (48). These interactions were conserved with other antagonists as SB-674042 and EPMA (49, 50). In 2016, the first structure of OX1R was obtained by the same group (51). The major difference between OX1R and OX2R structures was the presence, in the N-terminal extracellular domain, of two-turn α-helix connected with a linker to TM1 (**Figure 3**). This α-helix could play a role in the interaction with the two native peptides (51). The structure identification of the complex OX1R-suvorexant revealed that the two binding pockets (in OX1R and OX2R) were very similar showing that N318^6.55^, H344^7.39^ and also P123^3.29^ (**Figure 3**), however this last residue was not identified in OX2R as contact residue, were in interaction with antagonist molecules in OX1R (51). These data confirmed several structure-function relationship studies demonstrating that the alanine substitution of K120^3.26^, P123^3.29^, Y124^3.30^, Q126^3.32^, A127^3.33^, W206^5.29^, Y215^5.38^, F219^5.42^, Y311^6.48^, N318^6.55^, F340^7.35^, T341^7.36^, H344^7.39^, W345^7.40^ and Y348^7.43^ OX1R residues (**Figure 3**) had a deleterious impact on ligand interactions (21, 52). The determination of OX1R and OX2R structures allowed to analyze interactions between receptors and antagonists, to study the antagonist selectivity and to design new antagonist molecules (48). Until now, only X-ray structures of OX1R or OX2R complexed to antagonists including DORA or SORA were studied (48). To circumvent the absence of receptor structure bound to agonists, various studies using 3D-modeling associated to molecular dynamic (MD) were produced. One of these models based on homology modeling using OX2R structure, NTSR1 (neurotensin receptor 1) and the chemokine receptor CXCR4 structures, associated to OxA docking calculation revealed the presence of two binding modes named TM5-mode and TM7-mode related to the ligand interaction predictions with the TM5 and TM7 (53). It can be noted that the TM5-mode was compatible with mutagenesis experiments indicating that L11 and L15 could interact with ECL2 (21, 22). In contrast, other OX1R and OX2R 3D models complexed to OxB were produced by homology modeling associated to MD and mutagenesis data (22). These models indicated that the 11-28 sequence of OXB interacted with the orthosteric site of OX2R and OX1R (22). Our group has also produced a OX1R 3D model (**Figure 3**) based on homology modeling with X-ray structure of OX2R-suvorexant complex, associated to MD and mutagenesis data (21). This model shown that the C-terminal domain of OxB which adopted a helical conformation (sequence 21-27) was in contact with the orthosteric site (**Figure 3**) of the receptor involving van der Walls interactions, various hydrogen bonds with Y224^5.47^, N318^6.55^, T341^7.36^ and W345^7.40^ and ionic bond with E184 (21). Moreover, MD analysis shown that this model adopted an « active » conformation (**Figure 3**) mediated by an outward swing of TM6 and TM2 associated to sliding of TM3, essential for interaction with G protein transducer as recently demonstrated for OX2R in the presence of OxB (23). Our OX1R-OxB 3D model was close (root mean square distance (rmsd)=2.316) to the very recent structure of OX2R-OxB (**Figure 3**) (23). Indeed, the structure of the complex formed by OX2R, OxB or small molecule agonist 3′-(N-(3-(2-(2-(2H-1,2,3-triazol-2-yl)benzamido)ethyl)phenyl)sulfamoyl)-4′-methoxy-N,N-dimethyl-[1,1′-biphenyl]-3-carboxamide or compound 1), and mini Gq protein (**Figure 3**) was identified by single-particle cryo-electron microscopy (cryo-EM) (23). It should be noted that only the 20-28 sequence (C-terminal domain) of OxB was resolved in this study (**Figure 3**). The major difference as compared to previous OX2R-antagonist structures, was associated to a reorganization of TMs, (TM5 to TM7) displaying to conformational changes associated to the classical scheme of GPCR activation (23). The swinging outward movement of TM6 and TM2 (**Figure 3**) allowed the insertion of α5-helix of Gα_q_ subunit in the OX2R core (**Figure 3**). This study indicated that the masterswitches involved in GPCR activation as DRY (DRWY in OX2R), NPxxY and I/VPF motifs were well-rearranged (23). Analysis of interactions between OX2R and C-terminal part of OXB, revealed the participation of Q134^3.32^, F227^5.42^, T231^5.46^, I320^6.51^, N324^6.55^, Y343^7.32^, F346^7.35^, H350^7.39^ and Y354^7.43^ residues some of which identified by directed mutagenesis in OX1R played a major role in OxB binding (21, 23). The comparison between the OX2R-OxB/compound 1-miniGq complex structure to OX2R-antagonist complexes structure revealed the key role of Q134^3.32^ residue which would facilitate the GPCR activation transition (23).

## Orexins/OX receptors system signaling pathways

The biological effect of orexins in CNS and PNS (**Figure 1**) were mediated by interaction between orexins and their two receptors which were coupled canonically to the Gq protein (54). The binding of OxA or OxB to OX1R or OX2R induced a transmembrane helix movements leading to an active conformational state of receptors which facilitates the coupling to the heterotrimeric Gq protein (**Figure 2**). The heterocomplex Gq protein/OX1R or Gq protein/OX2R promoted the exchange of GDP for GTP on the αq subunit leading to its dissociation from activated receptors but also from β/γ subunits (55). GTP-bound αq subunit modulated the phospholipase C (PLC) activity which catalyzed membrane phosphatidylinositol-4,5-bisphosphate (PIP2) hydrolysis into inositol-1,4,5-trisphosphate (IP_3_) and diacyl glycerol. The binding between IP_3_ and their receptors induced the activation of Ca^2+^ ion channel located on endoplasmic reticulum triggering a transient Ca^2+^ release in cytoplasm (**Figure 2**) (55). This strong pulse of cytoplasmic Ca^2+^ promoted various intracellular enzymatic cascades responsible of the final biological actions of orexins (55, 56). Although, main orexin actions were mediated by intracellular Ca^2+^ release, some signaling pathways encompassing MAPK (mitogen-activating protein kinases) -Erk1/2 (extracellular signal-regulated kinases), cAMP, JNK (c-JUN N-terminal kinases) and PI3K (Phosphoinositide 3-kinase)-Akt were also activated by orexins (**Figure 2**) (54–56). More recently, our group has identified a new signaling pathway activated by orexins in cancer cells (57). The sequence analysis of OX receptors demonstrated the presence of two ITIM (immunoreceptor tyrosine-based inhibitory motif) sites in the transmembrane (TM) domains TM2 and TM7 of the receptor (57, 58). These ITIM sequences contain one tyrosine residue which was phosphorylated by src kinases when receptors were activated by orexins (57, 58). This phosphorylation led to the recruitment of the tyrosine-protein phosphatase non-receptor type 11 (SHP2) followed by the activation of p38 mitogen-stress protein kinase *via* RAS/MAPK signaling pathways, the translocation of Bax into the mitochondria, the cytochrome c releasing participating to the apoptosome formation involving the activation of caspase-3 and -7 and finally triggering cell apoptosis (**Figure 2**) (59).

## Anti-tumoral properties of orexins and receptor antagonist, almorexant

As previously described above, studies on pathophysiological roles of orexins and their receptors are focused on CNS (7) and mainly narcolepsy type I pathology (60). Moreover, a lot of studies also revealed the impact of orexins on addiction, neuro-inflammation associated to the microglia and neurodegenerative diseases including Alzheimer’s disease (11, 61, 62). More recently, the COVID-19 pandemic period has demonstrated the potential neuroinvasion and immunopathology of COVID-19 including the production of autoantibodies that can be associated with autoimmune diseases (63). Based on the correlation between autoantibodies production and Glasgow Coma Scale scores of patients, autoantibodies directed against OX2R which could antagonize OX2R activity, have been identified (63). Moreover, a negative correlation between anti-OX2R autoantibodies level in patients and low Glasgow Coma Scale scores has been observed (63) suggesting that these autoantibodies could interfere with central action of orexins in COVID-19 patients. In contrast, few studies are devoted to their physiological roles in PNS including impact on cardiovascular, genitourinary, digestive and neuroendocrine systems (11). Similarly, few studies were carried out to identify the role of orexins and their receptors in peripheral pathologies. However, it was demonstrated that orexins played an important role in the metabolic syndrome (13) and chronic inflammations including multiple sclerosis (64), septic shock (65) and ulcerative colitis (66). The identification of antitumoral role of orexins started in 2004 by experiments determining the impact of various neuropeptides including orexins, and various peptide hormones on the growth of colon cancer cell lines in standard condition of cell culture (67). If a large majority had no effect on cancerous cell growth, OxA and OxB were able to strongly reduced cell growth (67). These inhibitory properties were associated to induction of mitochondrial apoptosis (68). The mitochondrial apoptosis was mediated by OX1R and OX2R but only OX1R was expressed in human digestive cancers encompassing colorectal cancer (CRC), pancreatic ductal adenocarcinoma (PDAC), liver cancer (CHC), gastric cancer, esophagus cancer and cholangiocarcinoma (CCH) (13). It should be noted that OX1R was not expressed in corresponding healthy epithelia (68). OX1R was also expressed in human non-digestive cancers including prostate cancer (69), neuroblastoma (67), cortical adenomas (70), pheochromocytomas (71) and in endometrial carcinoma (72). Moreover, we have demonstrated that OX1R was expressed in human liver and lung metastasis from colon (68). The orexins/OX1R system-induced apoptosis activated by OxA or OxB (67) which was triggered by a new signaling pathway involving the SHP2 recruitment as mentioned in “introduction” section.

The CRC is the third commonest cancer worldwide and represents the third cause of cancer-related mortality (73). Like most cancers, CRC resulted in multiple genetic and epigenetic modifications leading various alterations of tumor suppressor genes involved in important cell signaling pathways (74). The first main line of treatment was surgery. However, in metastatic CRC, chemotherapy including 5-fluorouracil (5-FU), oxaliplatin and irinotecan which could be associated with immunotherapy in some cases (75). In 2011, we identified the expression of OX1R but not OX2R in 100% of colon tumors tested independently of grade state, location and genetic alterations (68). OX1R expression in colon cancer was early since OX1R was expressed dysplastic polyps which represented the pre-cancerous lesions leading to adenocarcinoma (76). This expression was recovered in 10 colon cancer cell lines including HT-29, LoVo, Caco-2 (68). In these cells, orexins induced a mitochondrial apoptosis *via* OX1R which was reverted by using SHP2 inhibitors as NSC-87877 and/or by using P38 inhibitor as PD169316 (68). Functional OX1R was always expressed in HT-29 cells resistant to 5-FU treatment in which OxA induced mitochondrial apoptosis (68). *In vivo*, subcutaneous injection of HT-29 or LoVo colon cancer cells in nude mice induced solid tumor development in few weeks. Daily intraperitoneal (ip) injection of OxA strongly reduced the tumor volume in mice (68). Moreover, the OxA treatment on established xenografted tumors (about 200-300 mm^3^) in nude mice were also strongly reduced tumor growth, indicating the anti-tumoral ability of OxA in well-implanted tumors. Histologic analysis of these tumors indicated that the OX1R expression was maintained during tumor development and that OxA treatment induced important apoptosis outbreaks in these tumors (68). It should be noted that endogenous OxA had no intrinsic action on tumor development because: 1) no OxA/OxB peptides were detected in the tumor environment (68); 2) the circulating level of OxA was very low (about 60 pM) does not allow OX receptors activation which displayed a 5-10 nM range of affinity (77); and 3) xenografted tumors developed from colon cancer cell line which does not express OX1R, shown a similar development kinetic that colon cancer cells expressing OX1R (68).

The PDAC which represents about 90% of exocrine pancreatic cancers is the tenth most common cancer and the fifth in terms of mortality (78). This cancer has a poor prognosis with a 5-year survival rate of about 8-10% (79). Prospective studies indicated that PDAC display the second cause of cancer-related deaths in 2030 (80). At this time, risk factors associated with the development of PDAC was not clearly established although classical risk factors were suggested as smoking, obesity, age and inflammation (81). As most cancers, epigenetic and genetic deregulation were observed as Kras mutation observed in >90% for PDAC (82). PDAC was a very aggressive cancer which rapidly induced metastasis associated to the poor prognosis of this cancer (79). Only 20% of PDAC were surgically resectable often associated to neo-adjuvant treatment (83). However, the level of relapse was particularly high (>70%). At metastatic stage, PDAC was treated by chemotherapy including gemcitabine, Nab-paclitaxel and FOLFIRINOX (folinic acid, 5-FU, irinotecan and oxaliplatin). Frequently, a chemoresistance associated to metabolic reprogramming appeared (84). OX1R but not OX2R was widely expressed in PDAC (>90% of tested tumors) (85). As observed in CRC, this expression was independent of the tumor grade, gender, patient age, etc. OX1R was also expressed in PDAC pre-neoplastic lesions named PanIN (pancreatic intraepithelial neoplasia) (85). Contrary to CRC, only few PDAC cell lines were available, one of them AsPC-1 cell line expressed OX1R. In these cells, OXA induced a mitochondrial apoptosis mediated by the SHP2 signaling pathway (85). OxA was also able to trigger SHP2-dependent apoptosis in cultured fresh slices of PDAC isolated from patient (85). In preclinical mouse model where mice were xenografted with AsPC-1 cell line or PDAC cells isolated from patient, OxA reduced tumor volume (85). In contrast, in HPAF-II cell line which does not express OX1R, OxA treatment did not have any effect (85). The OxA-dependent tumor volume reduction resulted of cell apoptosis in tumor as confirmed by histological analysis. It should be noted that OxA induced also anti-tumoral actions in xenografted tumors resistant to gemcitabine or NAB-paclitaxel which represent the “gold-standard” treatment of PDAC (86). As previously mentioned above, a lot of OX1R and OX2R antagonist were developed to treat insomnia, surprisingly, DORA compounds as almorexant and suvorexant were able to induce cell apoptosis in AsPC-1 cell line although these compounds antagonized the Ca^2+^ release triggered by OxA (85). Ip injection of almorexant in nude mice xenografted with AsPC-1 cells reduced the tumor volume (85). It seems that almorexant and also suvorexant behaved as agonist toward SHP2 signaling pathway which is dependent of β/γ subunits but as an antagonist of the Ca^2+^ release signaling pathway which is dependent of α_q_ subunit. These observations are in contradiction with the dogma GPCR activation mechanism indicating that antagonist stabilizes inactive state of receptors and agonist stabilizes its active state (87). In case of OX1R, almorexant would block the activation of α_q_ subunit but would allow to β/γ subunits to activate src kinases involved in ITIM sites phosphorylation of receptors. However, it is not clearly proven that activated GPCR release β/γ subunits in cytosol (88). Various hypothesizes have been developed including a complete dissociation between α_q_ and β/γ subunits, a sliding of β/γ subunits along αN helix of α_q_ subunit exposing the effector-interacting domains and/or the “clamshell model” where α_q_ and β/γ subunits open up to expose the interacting domains (88, 89). Almorexant could behave as partial antagonist/partial agonist toward Gq protein by inactivation of α_q_ subunit but allowing to β/γ subunits to recruit and to activate src kinases. Additional experiments using BRET technology with fluorescent/luminescent probes including receptors and mini-Gq protein should be designed to investigate the impact of agonist/antagonist in recruitment/dissociation mechanism between OX1R and Gq subunits.

## Conclusion

In less than 25 years, the orexins/OXR system has been discovered and its major pathophysiological roles in CNS have been identified. Moreover, its mechanism of action was deciphered and the development of a wide range of pharmacological molecules associated to structural studies have been designed. However, much remained to be done including the pathophysiological role of orexins and their receptors in PNS. Recent studies demonstrated the anti-inflammatory and anti-tumoral actions of orexins paving the way for the further development in human health.

## Author contributions

AC has written the manuscript. PN, VG, and TV have reviewed, modified and revised the manuscript. All authors have read, edited and approved the final version of manuscript.

## Funding

Our work was supported by the “Institut National de la Santé et de la Recherche Medicale” (INSERM), the “Université Paris Cité”, The “Institut National du Cancer (INCA)” [PAIR Pancreas, grant number N° PAN18-045] and the “Ligue Nationale Contre le Cancer” [grant numbers R16020HH, GB/MA/CD/EP-12062].

## Acknowledgments

Molecular graphics presented in **Figures 2**, **3** were performed with UCSF Chimera, developed by the Resource for Biocomputing, Visualization, and Informatics at the University of California, San Francisco, with support from NIH P41-GM103311 (https://www.cgl.ucsf.edu/chimera/).

## Conflict of interest

The authors declare that the research was conducted in the absence of any commercial or financial relationships that could be construed as a potential conflict of interest.

## Publisher’s note

All claims expressed in this article are solely those of the authors and do not necessarily represent those of their affiliated organizations, or those of the publisher, the editors and the reviewers. Any product that may be evaluated in this article, or claim that may be made by its manufacturer, is not guaranteed or endorsed by the publisher.
